# The predictive validity of a situational judgement test and multiple-mini interview for entry into postgraduate training in Australia

**DOI:** 10.1186/s12909-016-0606-4

**Published:** 2016-03-09

**Authors:** Fiona Patterson, Emma Rowett, Robert Hale, Marcia Grant, Chris Roberts, Fran Cousans, Stuart Martin

**Affiliations:** Work Psychology Group, 27 Brunel Parkway, Pride Park, Derby, DE24 8HR UK; Psychology Department, University of Cambridge, Downing Street, Cambridge, CB2 3EB UK; Formerly General Practice Education & Training (GPET), Canberra, Australia; Sydney Medical School - Northern, Hornsby Ku-ring-gai Hospital, Palmerston Road, Hornsby, NSW 2077 Australia

**Keywords:** General Practice, Postgraduate, Postgraduate selection, Reliability and validity, Selection, Situational judgement test, SJT, Multiple-mini interview, MMI

## Abstract

**Background:**

Evidence for the predictive validity of situational judgement tests (SJTs) and multiple-mini interviews (MMIs) is well-established in undergraduate selection contexts, however at present there is less evidence to support the validity of their use in postgraduate settings. More research is also required to assess the extent to which SJTs and MMIs are complementary for predicting performance in practice. This study represents the first longitudinal evaluation of the complementary roles and predictive validity of an SJT and an MMI for selection for entry into postgraduate General Practice (GP) specialty training in Australia.

**Methods:**

Longitudinal data was collected from 443 GP registrars in Australia who were selected into GP training in 2010 or 2011. All 17 Regional Training Providers in Australia were asked to participate; performance data were received from 13 of these. Data was collected for participants’ end-of-training assessment performance. Outcome measures include GP registrars’ performance on the Royal Australian College of General Practitioners (RACGP) applied knowledge test, key feature problems and an objective structured clinical exam.

**Results:**

Performance on the SJT, MMI and the overall selection score significantly predicted all three end-of-training assessments (*r* = .12 to .54), indicating that both of the selection methods, as well the overall selection score, have good predictive validity. The SJT and MMI provide incremental validity over each other for two of the three end-of-training assessments.

**Conclusions:**

The SJT and MMI were both significant positive predictors of all end-of-training assessments. Results provide evidence that they are complementary in predicting end-of-training assessment scores. This research adds to the limited literature at present regarding the predictive validity of postgraduate medical selection methods, and their comparable effectiveness when used in a single selection system. A future research agenda is proposed.

## Background

The proportionate effectiveness of selection methods for entry into postgraduate medical training has been a relatively under-researched topic internationally [1–4]. As with all selection methodologies, various psychometric criteria must be satisfied to ensure that a given postgraduate medical selection system is fair and robust, including standardisation, reliability and validity [5–7]. Faced with limited training positions and a high volume of applicants, medical selection has traditionally relied on academic attainment as primary selection criteria in admission systems [8]. However, there is a growing recognition in the literature that other important non-academic attributes and skills must be present from the start of training in order to become a competent clinician [9]. Given that medical selection systems globally are increasingly implementing several selection methods in combination (targeting both the academic and non-academic attributes required of clinicians), there is a need to evaluate the relative and complementary roles of, and value-added by, selection methods in predicting desired outcome criteria, which to date is lacking in the research literature [2].

Internationally, extensive literature documents the reliability, validity and stakeholder acceptability of situational judgement tests (SJTs) as measures of non-academic ability across a range of occupations, including in the context of medical selection [2, 10–14]. However, although evidence regarding the construct validity and reliability of SJTs exists at the postgraduate level for selection into UK General Practice (GP) [3, 15, 16], there is limited extant research internationally regarding the predictive validity of SJTs for selection into postgraduate specialty training.

One high volume postgraduate specialty that has recently incorporated non-academic assessment at the point of selection is Australian GP training. The current selection system was implemented nationally in 2011 following a successful pilot in 2010, and comprises an SJT and a multiple-mini interview (MMI). The standardised results of the SJT and MMI determine an applicant’s overall selection score. Applicants’ overall selection score and geographic training region preference are used to determined if the applicant can be shortlisted for subsequent local selection processes.

**Fig. 1:**
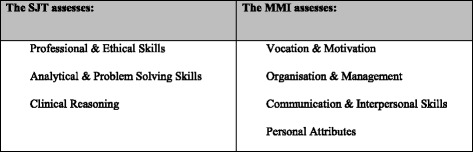
Attributes assessed by the Australian GP selection methods

The selection process targets seven core attributes (detailed in Fig. 1), which were criterion-matched against the competencies identified as important for entry-level GP registrars in the domains of practice defined by the Royal Australian College of General Practitioners (RACGP) and the Australian College of Rural and Remote Medicine (ACRRM).

The Australian GP selection process does not include an explicit measure of academic attainment at the point of selection, unlike more traditional selection systems. However, completion of a primary medical qualification is a requirement for eligibility, therefore academic attainment is a prerequisite at the point of selection. In addition, academic attainment tends to be relatively homogenous in trainee physicians, therefore differentiating between applicants for postgraduate medical education predominantly on the basis of academic achievement is challenging and likely to be error-prone [17–19]. Instead, as outlined in Fig. 1, related attributes such as clinical reasoning and problem solving are assessed via an SJT. Preliminary, cross-sectional evidence of the reliability and concurrent validity of the selection system has been demonstrated [20], however to date longitudinal data has not been collected to assess the validity of the selection system for predicting performance in end-of-training assessments. Moreover, the complementary roles of the SJT and MMI have yet to be assessed. In any multi-method selection system, it is important that each method has added value (i.e., assesses something different to the other tools in the system) in order to ensure that efficiency and cost-effectiveness is maximised. Therefore, in the present study we posed the following research questions:

1. *What is the predictive validity of the SJT, the MMI, and the overall selection score for performance on end-of-training assessments in Australian GP training?*
2. *What are the incremental validities of the SJT and the MMI for predicting performance on end-of-training assessments in Australian GP training?*

## Method

### Participants

Selection data was collected from participants who took part in the 2010 and 2011 selection process into Australian GP training, which comprised both the SJT and the MMI. Participants provided their consent at the point of selection into GP training for their data to be used for research purposes. In 2010, this new selection process was piloted by three Regional Training Providers (*N* = 345) and in 2011 this selection process was used nationally across Australia (*N* = 1335).

End-of-training assessment scores were requested from all 17 Regional Training Providers, and received from 13 of these. From this data, it was possible to match the selection and end-of-training data for *N* = 443 registrars. Table 1 provides the sample and entire population’s demographics, which shows that our sample is consistent with the demographic breakdown of the entire population.

**Table 1:** Demographics

	N	2010 Cohort	2011 Cohort	Mean age at time of selection	Women	Men	Australian medical graduates (inc. NZ)	International medical graduates
Overall sample	1680	345 (20.54 %)	1335 (79.46 %)	33 (SD 7.47)	1058 (62.98 %)	622 (37.02 %)	1027 (61.13 %)	653 (38.87 %)
Matched Sample	443	114 (25.73 %)	329 (74.27 %)	31.61 (SD 6.51)	267 (60.27 %)	176 (39.73 %)	289 (65.24 %)	154 (34.76 %)

### Procedure

A retrospective longitudinal design, using previously validated methods [2, 10–14], was used to evaluate the selection data’s relationship with end-of-training assessment scores.

Analyses were conducted using SPSS 22.0 for Windows. Pearson product–moment correlations were used to assess the association between all selection and end-of-training assessments, and hierarchical regression analyses examined the predictive power of the selection methods. Missing data were deleted pairwise to maximise the available sample size for each analysis.

### Selection methods

#### Situational judgement test

**Fig. 2:**
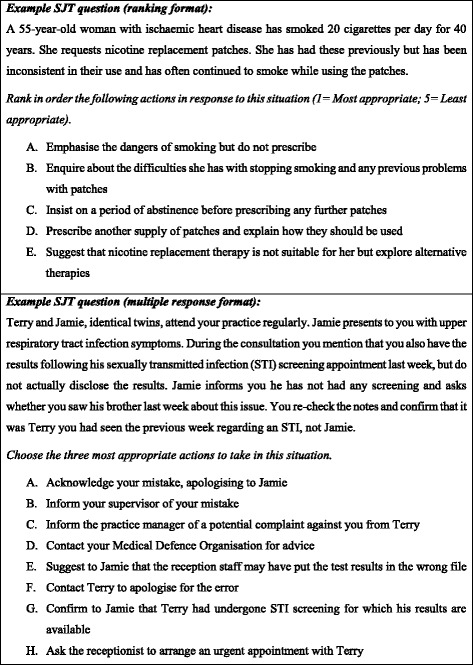
Example SJT Questions

The SJT is a low fidelity computer-delivered examination which is completed under invigilated conditions. The test comprises 50 questions and applicants have two hours to complete the test. Two response formats are used: ranking and multiple response (see Fig. 2). This SJT has been found to have high internal reliability (Cronbach’s alpha = .91) [21].

#### Multiple-mini interview

**Fig. 3:**
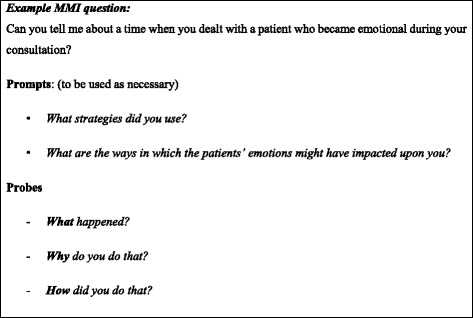
Example MMI Question

The MMI rotates applicants between six, 10 min interview stations. Applicants have two minutes to read the question before entering the interview room, then eight minutes to answer the question from the interviewer, in a face-to-face context. Interviewee responses are then probed further by the interviewer. An example MMI question is provided in Fig. 3. Each interviewer gives the applicant a score out of seven based on standardised criteria. This specific MMI has been found to have high internal reliability [21] (mean Cronbach’s alpha = .76).

The SJT and MMI are each weighted as 50 % of the overall selection score.

### End-of-training assessments

The outcome measures for this study were end-of-training assessment scores for the final RACGP Fellowship assessment, consisting of three invigilated assessments;

#### Applied knowledge test

The applied knowledge test is a multiple-choice examination, which includes 150 clinically-based questions delivered via computer over three hours.

#### Key feature problems

This is a computer delivered examination paper that assesses clinical decision making skills. The 26 ‘key feature problems’ each consist of a clinical case scenario followed by questions that focus only on those critical steps. Candidates are required to type short responses or choose from a list of options provided and the assessment lasts for three hours.

#### Objective structured clinical exam

This is a four hour high fidelity clinical performance assessment of applied knowledge, clinical reasoning, clinical and communication skills, and professional behaviours in the context of patient consultations and peer discussions. They are combined of 14 clinical cases of either short eight minutes or long 19 min stations, including rest stations.

For each of these assessments, GP registrars were able to complete the assessment multiple times. For the purposes of this study, the applicants’ ‘best’ score on each assessment was utilised. The reliability of each of the end-of-training assessments could not be calculated from the data collected, and was not readily accessible online at the time of publication.

## Results

### Descriptive statistics

Matched data was available from 443 registrars. All variables in the study showed normal distributions, with the exception of the SJT which had a slight negative skew, as is typical of SJT score distributions [20, 22, 23]. Skewness of the SJT score distribution was assessed, and was within acceptable limits (−1.28). As such, parametric analyses were run on all variables, as previous research has suggested that apart from in instances of extreme skew, parametric analyses are more powerful and robust [24, 25].

Raw scores from each of the three end-of-training assessments were converted into percentage scores, to enable direct comparison of results between assessments. Table 2 details the descriptive statistics for scores on the selection methods and end-of-training assessments.

**Table 2:** Descriptive statistics for selection methods and end-of-training assessments

	N	Min	Max	Mean	SD
Selection Methods					
SJT Weighted Score	443	37.09	57.39	51.10	3.22
MMI Weighted Score	443	39.35	60.58	51.00	4.11
Overall Selection Score	443	82.84	114.56	102.10	6.13
End-of-training Assessments					
Applied Knowledge Test Best Score	441	59.29	94.48	78.14	6.13
Key Feature Problems Best Score	435	48.80	82.86	68.05	5.91
Objective Structured Clinical Exam Best Score	395	58.61	90.21	75.18	6.31

A significant correlation (*p* < .001) was found between scores on the SJT and MMI in the population as a whole, as well as in the matched sample (*r* = .53, *N* = 1594; *r* = .39, *N* = 443 respectively). The slightly smaller correlation in the matched sample is to be expected given the likely restriction of range inherent in successful applicants’ selection scores.

### Predictive validity of the selection methods

Table 3 presents the correlations between the selection methods and the end-of-training assessments. Results showed that both the SJT and MMI, as well as the overall selection score, are significantly correlated with performance on all end-of-training assessments (*r* values ranging from .12 to .54; *p* < .05 to *p* < .001).

**Table 3:** Pearson’s correlations between selection methods and end-of-training assessments

*N* = 443	1.	2.	3.	4.	5.
Selection Methods					
1. SJT Weighted Score	–				
2. MMI Weighted Score	.39***	–			
3. Overall Selection Score	.79***	.88***	–		
End-of-training Assessments					
4. Applied Knowledge Test Best Score	.14**	.12*	.15***	–	
5. Key Feature Problems Best Score	.24***	.20***	.26***	.54***	–
6. Objective Structured Clinical Exam Best Score	.44***	.46***	.54***	.33***	.45***

* *p* < .05, ** *p* < .01, ***p < .001

The SJT and MMI both showed a particularly strong correlation with the objective structured clinical exam (*r* = .44 and *r* = .46 respectively, both *p* < .001), which is likely to reflect the similarity in content between these two assessments; i.e., both the SJT and MMI have been designed to assess non-academic attributes. While the SJT and MMI correlated at a similar level with both the applied knowledge test (*r* = .14, *p* < .01 and *r* = .12, *p* < .05, respectively) and the key feature problems (*r* = .24, *r* = .20 respectively, both *p* < .001), correlations were substantially smaller than the correlations between the SJT and MMI, and the objective structured clinical exam.

### Incremental validity of the SJT and MMI

Hierarchical regression analyses were conducted to ascertain the extent to which the SJT and MMI explain significant added value (incremental validity) over and above each other, for predicting scores on all three end-of-training assessments. Results are shown in Table 4.

**Table 4:** Hierarchical regression analysis of the SJT and MMI with end-of-training assessments

		Unstandardised b	SE b	β	t	F	R-sq	ΔR-sq
Applied Knowledge Test *N* = 441								
Additional variance explained by SJT over MMI								
Step 1	MMI	.18	.07	.12	2.49	6.21	.01*	
Step 2	MMI	.11	.08	.07	1.45	5.59	.03*	.01*
	SJT	.22	.10	.11	2.22			
Additional variance explained by MMI over SJT								
Step 1	SJT	.27	.09	.14	3.01	9.07	.02*	
Step 2	SJT	.22	.10	.11	2.22	5.59	.03	.01
	MMI	.11	.08	.07	1.45			
Key Feature Problems *N* = 435								
Additional variance explained by SJT over MMI								
Step 1	MMI	.29	.07	.20	4.31	18.58	.04***	
Step 2	MMI	.19	.07	.13	2.61	16.36	.07***	.03***
	SJT	.34	.09	.19	3.69			
Additional variance explained by MMI over SJT								
Step 1	SJT	.43	.09	.24	5.06	25.57	.06***	
Step 2	SJT	.34	.09	.19	3.69	16.36	.07**	.02**
	MMI	.19	.07	.13	2.61			
Objective Structured Clinical Exam *N* = 395								
Additional variance explained by SJT over MMI								
Step 1	MMI	.71	.07	.46	10.22	104.37	.21***	
Step 2	MMI	.52	.07	.34	7.37	81.15	.29***	.08***
	SJT	.60	.09	.31	6.78			
Additional variance explained by MMI over SJT								
Step 1	SJT	.85	.09	.44	9.75	95.07	.20***	
Step 2	SJT	.60	.09	.31	6.78	81.15	.29***	.10***
	MMI	.52	.07	.34	7.37			

*p < .05, **p < .01, ***p < .001

The SJT explains a significant amount of additional variance, over the MMI, in the applied knowledge test, the key feature problems and the objective structured clinical exam scores (1 %, 3 % and 8 % respectively). The MMI explains a significant amount of additional variance, over the SJT, in the key feature problems and the objective structured clinical exam scores (2 and 10 % respectively).

## Discussion

This study provides longitudinal data to advance the relative dearth of research regarding the predictive validity of selection methods in postgraduate medical settings. This is the first study to explore the relative predictive validity of, and value added by, an SJT and an MMI within a single postgraduate specialty selection system. Our results show that the SJT and MMI are significantly correlated with end-of-training assessment performance, indicating that each selection method, and the overall selection score, has good longitudinal predictive validity. Regression analyses indicate that these relationships are significantly predictive of performance across all end-of-training assessments.

There is a moderate correlation between the SJT and MMI, suggesting that these selection methods have both common and independent variance, and therefore that each method offers a unique contribution to the selection system. Both the selection methods explain significant additional variance over each other in predicting performance on the end-of-training assessments. The SJT explains additional variance over the MMI for performance on the applied knowledge test, but the opposite is not true. Practically, this means that the selection model with the best predictive validity of end-of-training assessment performance is a combination of both the SJT as MMI as both methods contribute incremental validity over and above the other in predicting training outcomes.

These are important findings as this is the first longitudinal study exploring the predictive validity of medical postgraduate selection methods in Australia. The results have relevance internationally, as they suggest that the combination of an SJT and an MMI is effective in identifying applicants who go on to perform well in assessments at the end of specialty medical training. These findings progress the current literature regarding the relative contributions of different selection methodologies when methods are used in combination, which a recent systematic review indicated is lacking at present [2].

The SJT and MMI show a particularly strong correlation with the objective structured clinical exam. The strong correlation between the MMI and objective structured clinical exam is likely to reflect the similarities between these two assessments, for example that they are both face-to-face (high fidelity) and assess an individual’s ability to communicate effectively and respond to a question or situation in an appropriate way. Importantly, although the SJT is a low fidelity written assessment, the positive correlation with the objective structured clinical exam is especially encouraging, as compared to the MMI, a text based SJT is significantly less resource intensive to deliver and can be machine marked. Comparatively lower correlations were found between the selection methods and the applied knowledge test, which is expected given that the applied knowledge test is a measure of declarative knowledge and could be considered the least consistent assessment with the selection methods in terms of underlying constructs being measured; we would not expect an SJT (designed to assess non-academic constructs and interpersonal skills) to predict performance on a highly cognitively loaded criterion [26]. We would, however, expect an SJT to predict performance on criterion-matched outcomes such as interpersonal skills and patient care [26], as assessed by the objective structured clinical exam and the key feature problems test.

### Implications

Considering priorities for a future research agenda for evaluating the predictive validity of selection into Australian GP training, it would be prudent to gather criterion-matched in-training (i.e., mid-GP training) performance data, and if possible, gather performance data from registrars once they enter practice. This would allow analysis of the predictive validity of the selection methods throughout GP training and beyond, and such data is lacking in the medical selection research at present [2]. This is important as indicators of competence, and selection methods, have been found to be differentially predictive of performance at different stages of medical training [2, 26, 27]. Specifically, non-academic measures have been found to be more predictive in the later stages of medical education and training, for example, conscientiousness has been identified as a predictor of success in undergraduate training, but may actually hinder aspects of performance in clinical practice [27]. As such, different selection methods may predict differently at different stages, for example, an SJT may be less predictive of academic performance in the early years of training, but significantly more predictive of performance outcomes once trainees enter clinical practice [28, 29]. Thus, it would be beneficial for future research to follow the current cohort of applicants once they enter independent clinical practice.

### Limitations

As this is the first analysis of predictive validity, we have adapted a conservative approach to data analysis and have not corrected for restriction of range in the present study; therefore these results are likely to have underestimated the magnitude of relationships between selection methods and performance on end-of-training assessment. However, future analysis of more longitudinal data (i.e., mid-GP training, into the consultant role, and beyond) may benefit from restriction of range analysis, as the pool of applicants is likely to diminish at each stage, thus increasing range restriction which serves to supress the magnitude of the predictive validity coefficients. Another limitation of this study is the relatively small sample size, therefore it would be beneficial to conduct further research on a larger sample size.

The reliability of each of the end-of-training assessments could not be calculated from the data collected, and were not readily accessible online at the time of publication. As such, it is difficult to determine the reason for the comparatively weak correlations between the SJT and MMI, and the applied knowledge test. However, it should be noted that the SJT and MMI are designed to target different constructs when compared to the applied knowledge test, so these results are expected to some extent.

## Conclusions

This study represents the first longitudinal analysis of the predictive validity of the methods for selection into Australian General Practice training. The SJT and MMI were significant positive predictors of all three end-of-training assessments. Results show that the two selection methods are complementary as they both explain incremental variance over each other for end-of-training assessment scores. This research therefore adds to the relatively sparse literature at present regarding the predictive validity of postgraduate medical selection methods, and their comparable effectiveness when used in a single selection system. Future research would benefit from more longitudinal research with criterion-matched outcomes, across the duration of GP training, and once they enter independent clinical practice.

## Ethics statement

Participants provided their consent at the point of selection into GP training for their data to be used for research purposes. Ethical approval for this specific study was granted via the University of Sydney.

## Abbreviations

SJT: situational judgement test
MMI: multiple-mini interview
GP: general practice
RACGP: Royal Australian College of General Practitioners
ACRRM: Australian College of Rural and Remote Medicine
